# Plate-Based Respirometry to Assess Thermal Sensitivity of Zebrafish Embryo Bioenergetics *in situ*

**DOI:** 10.3389/fphys.2021.746367

**Published:** 2021-09-21

**Authors:** Erik Rollwitz, Martin Jastroch

**Affiliations:** Department of Molecular Biosciences, The Wenner-Gren Institute, Stockholm University, Stockholm, Sweden

**Keywords:** extracellular flux, zebrafish, embryo, oxygen consumption, temperature, proton leak, mitochondria

## Abstract

Oxygen consumption allows measuring the metabolic activity of organisms. Here, we adopted the multi-well plate-based respirometry of the extracellular flux analyzer (Seahorse XF96) to investigate the effect of temperature on the bioenergetics of zebrafish embryos (*Danio rerio*) *in situ*. We show that the removal of the embryonic chorion is beneficial for oxygen consumption rates (OCR) and penetration of various mitochondrial inhibitors, and confirm that sedation reduces the variability of OCR. At 48h post-fertilization, embryos (maintained at a routine temperature of 28°C) were exposed to different medium temperatures ranging from 18°C to 37°C for 20h prior OCR measurement. Measurement temperatures from 18°C to 45°C in the XF96 were achieved by lowering the room temperature and active in-built heating. At 18°C assay temperature, basal OCR was low due to decreased ATP-linked respiration, which was not limited by mitochondrial power, as seen in substantial spare respiratory capacity. Basal OCR of the embryos increased with assay temperature and were stable up to 37°C assay temperature, with pre-exposure of 37°C resulting in more thermo-resistant basal OCR measured at 41°C. Adverse effects of the mitochondrial inhibitor oligomycin were seen at 37°C and chemical uncouplers disrupted substrate oxidation gradually with increasing assay temperature. Proton leak respiration increased at assay temperatures above 28°C and compromised the efficiency of ATP production, calculated as coupling efficiency. Thus, temperature impacts mitochondrial respiration by reduced cellular ATP turnover at lower temperatures and by increased proton leak at higher temperatures. This conclusion is coherent with the assessment of heart rate, an independent indicator of systemic metabolic rate, which increased with exposure temperature, peaking at 28°C, and decreased at higher temperatures. Collectively, plate-based respirometry allows assessing distinct parts of mitochondrial energy transduction in zebrafish embryos and investigating the effect of temperature and temperature acclimation on mitochondrial bioenergetics *in situ*.

## Introduction

All species require temperature adaptation of their bioenergetics for maintenance of metabolism and life. Low temperatures decrease cellular metabolism of ectothermic species as they mainly lack thermogenic capabilities for maintaining constant body temperatures. Hence, ectotherms must balance temperature-sensitive cellular energy production and consumption to ensure physiological functions, such as growth, activity, and reproduction. While water temperatures fluctuate naturally due to weather and seasons, temperature sensitivity becomes increasingly important in the long-term scope of global warming, where aquatic ecosystems and thus ectotherms are challenged. The global mean ocean surface temperature is expected to increase by 1.8–4°C by the end of this century (Rhein et al., 2013), which will presumably elevate ocean warming, acidification, and hypoxia (Hoegh-Guldberg and Bruno, 2010). The effect of warming will depend on the thermal niche of the respective species. Species living below their thermal optimum will benefit from warm conditions, while others close to their thermal limit will suffer (Shephard et al., 2010; Eymann et al., 2020).

Many factors, such as physiological limitations, macromolecules, and genetic traits, need to be considered when looking at the plasticity of ectothermic (and endothermic) species in their response to changing environmental temperatures. An important physiological factor is aerobic scope; it represents the absolute difference between the maximum and standard rates of organismal aerobic metabolism (Gleeson, 1981; Halsey et al., 2018). In aquatic ectotherms, a decrease in aerobic scope is considered as the beginning of physiological thermal limitation on both ends of the thermal window, caused by a reduced capacity of the cardiovascular and pulmonary system to meet the increasing oxygen demand (Portner and Knust, 2007). The slowing of ventilation and circulation in the cold and the insufficient increase in the warm cause a mismatch between oxygen delivery and demand, which results in a limitation of thermal tolerance (Pörtner, 2001). Additional molecular constraints impact optimal function of organisms across a wide range of temperatures. Ectotherms in cold environments may increase the production of enzymes to compensate for decreased catalytic activity (Guderley, 2004). These modifications, also referred to as extrinsic factors, can occur relatively fast in response to environmental changes. Changes in temperature may also affect the structure and stability of proteins and enzymes, such as the loss of substrate binding affinity in teleost fish with increasing assay temperatures (Fields and Somero, 1998). Processes that aim to stabilize proteins, for example, by altering amino acid composition and secondary structures of proteins (Tattersall et al., 2012), occur at a slower pace during development or over generations, also referred to as intrinsic modifications (Travis et al., 1999; Fangue et al., 2009). Some genetic traits may shift expression to genes that provide better protection against the prevailing environmental conditions to alter the optimal thermal window of ectotherms. Further, behavioral changes and selection of microhabitats need to be considered when looking at ectothermic model organisms (Angilletta et al., 2009). Taken together, the response to thermal fluctuations is complex due to many factors. A general molecular mechanism or genetical program that enables aquatic ectotherms to maintain basic physiological function in a relative wide thermal spectrum remains elusive.

To measure thermal viability in aquatic ectotherms, several methodological approaches and model organisms with various thermal tolerance have been used, such as annelids (*Arenicola marina*) sipunculids (*Sipunculus nudus*), bivalves (*Ostrea edulis*), cephalopods (*Lolliguncula brevis*), mollusks (*Laternula elliptica*), crustaceans (*Maja squinado*), and teleost fish (*Fundulus heteroclitus*; Zielinski and Po, 1996; Pörtner and Zielinski, 1998; Urban and Silva, 1998; Sommer and Pörtner, 1999; Frederich et al., 2000; Schulte, 2015; Eymann et al., 2020). The range of parameters to judge thermal plasticity includes gene expression, enzyme activities, motility (e.g., swimming speed), and heart and metabolic rates (e.g., indirectly *via* oxygen consumption; Lahnsteiner and Mansour, 2012; Little et al., 2013; Ferreira et al., 2014; Veilleux et al., 2015; Pichaud et al., 2019).

From the experimental point of view, a good model organism has a sequenced genome and can be genetically manipulated, thereby offering the possibility to establish causality of molecular mechanisms.

The zebrafish (*Danio rerio*) is a vertebrate model used broadly in many disciplines ranging from developmental biology to drug discovery, offering an array of established tools ranging from genetic modification to standardized behavioral analysis. Thermal adjustments of its metabolism can be investigated over a large thermal window, as the zebrafish naturally inhabits freshwater with a wide temperature range of 16.5 to 34°C (Engeszer et al., 2007) in the tropics of South Asia. Previous studies measured metabolic rates as oxygen consumption of living zebrafish in response to different temperatures, using metabolic chambers/tanks (Little et al., 2013). To further understand the underlying mitochondrial mechanisms, it would be advantageous to isolate mitochondria and determine mitochondrial activities with Clark-type oxygen electrodes, as has been done in mollusks, for example, Kurochkin et al.(2009). The quantity and purity of isolated mitochondria, however, are limited for a small organism, such as the zebrafish. With new technologies, such as plate-based respirometry of the Seahorse extracellular flux analyzer, however, it may be possible to get insights into mitochondrial mechanisms and adaptations in response to thermal challenges by subjecting living organisms to the assay and measure oxygen consumption *in situ* (Divakaruni et al., 2014). A few studies demonstrated how to adapt the Seahorse system to the zebrafish and how to measure respiration of embryos. Stackley et al. investigated the change of embryonic bioenergetics over the course of early development at a standard temperature of 28.5°C, showing the increase of oxygen consumption rates (OCR) from 3 to 48h post-fertilization (hpf) in all respiratory parameters except proton leak (Stackley et al., 2011). The high individual variability of OCR (Souders et al., 2018) could be mitigated by sedation using tricaine (MS-222) pre-exposure (Raftery et al., 2017). Spheroid capture plates of the 24-well Seahorse system were used to determine the impact of the chorion on the respiratory responses to chemical uncoupler FCCP (Carbonyl cyanide-p-trifluoromethoxyphenylhydrazone) during development (Souders et al., 2018). Lee et al. (2019) curated Seahorse studies using whole zebrafish embryos, providing a list until 2019, where experimental conditions have been summarized to optimize the assay for testing toxins and pollutants (Lee et al., 2019). Plate-based respirometry is found in a few studies for testing the impact of toxins during early embryo development. For example, Shim and colleagues used 96-well Seahorse technology, enabling higher n-values to test the bioenergetic effects of triclosan, a synthetic antimicrobial agent commonly used in consumer goods (Shim et al., 2016).

In this paper, we applied the XF96 Seahorse extracellular flux analyzer platform to investigate the bioenergetic effects of various assay temperatures (from 18°C to 45°C) and the effect of pre-exposing the zebrafish embryos to temperatures ranging from 18°C to 37°C.

## Materials and Methods

### Animals

#### Maintenance

Fertilized eggs of wild-type (AB-strain) zebrafish (*Danio rerio*) were obtained from the Zebrafish core facility at Karolinska Institute after crossing under controlled conditions using breeding traps. Dividers were pulled at 6AM and eggs were collected for transport to Stockholm University. The eggs were kept in E3 medium (5mM NaCl, 0.17mM KCl, 0.33mM CaCl2, 0.4mM MgCl2, and 10^−5^% Methylene Blue, pH 7.2). The freshly laid eggs were kept at 28.5°C and picked up within 3h. During transport, the embryos were briefly exposed to approximately 22°C before returning to 28.5°C. Up to 200 embryos were maintained in one large petri dish (150mm×20mm, P5606, Sarstedt, Germany). Checking for dead embryos and medium replacement was done twice during the first experimental day and once in the morning of the second day (see Supplementary Figure for comprehensive description of the study design). The studies were approved by the Stockholm North Ethical Committee with the permit number 14049–2019.

#### Dechorionation

At 24h post-fertilization (24 hpf), the chorion was removed by a combination of enzymatic digestion and mechanical force using thin forceps. Embryos were transferred with a plastic Pasteur pipette from their large petri dishes to a beaker containing E3 medium. Pronase from *Streptomyces griseus* (SKU: 10165921001, Sigma-Aldrich, United States) was added at a concentration of 2mg/ml. The medium was swirled in a continuous movement of the beaker and the digestion states were checked microscopically for approximately 5min, or until the first embryos separated from their chorion. Additionally, the chorion integrity was checked by gently nudging the chorionated embryos with a pipet tip. The Pronase treatment was considered complete when the choria appeared soft. Embryo survival rates of nearly 100% after enzymatic digestion were achievable with careful treatment and immediate deactivation of Pronase activity, by shortly washing the embryos five times in Pronase-free E3 medium. Notably, solely the washing steps separated a fair amount of choria and embryos, while the residual individuals had to be separated with forceps. For this, the chorion of remaining embryos was removed by tearing the chorion with two forceps (Dumont no. 5) after transfer to a smaller petri dish (100mm x 20mm, P5606, Sarstedt, Germany). We improved the duration of chorion removal of ~350 embryos from 60 to 30min during the course of our studies. Chorion removal is also possible without Pronase treatment, but results in a significant number of crushed embryos (~15% in our hands), due to the mechanical force imposed on the embryos while tearing the rigid chorion with forceps. Furthermore, the time of chorion removal will increase about 3–5 times.

### Temperature Exposure

The embryos were checked for viability before exposing them to either 18°C, 23°C, 28°C, 33°C, or 37°C in sealed 50ml falcon tubes (62.547.254, Sarstedt, Germany) in a temperature-controlled water bath. Up to 70 embryos were transferred into the falcon tubes containing 50ml of E3 medium. The embryos were exposed to the respective temperature for 20h (see Supplementary Figure). We did not term temperature exposure “acclimation”, as we did not evaluate acclimation steady states. At 48 hpf, the zebrafish embryos were transferred to respirometric analysis.

### Microscopical Phenotyping

Embryos were transferred from the temperature incubation tubes into a petri dish (100mm multi 20mm, P5606, Sarstedt, Germany) for microscopy. The embryos were categorized into the phenotypes of normal, curved, and “other” (collection of very minor diverse phenotypes, e.g., showing edema) by visual inspection. Images were taken with the EVOS XL core microscope (Invitrogen, United States) at 4x magnification. Scale bars were added during post-processing with the aid of a Bürker counting chamber and the software AxioVision (release 4.8, Carl Zeiss, Germany).

### Protein and DNA Quantification

Embryo lysis, protein, and DNA quantification were performed to evaluate differences in biomass of the embryos in response to temperature pre-exposure.

#### Lysis of Zebrafish Embryos

A petri dish (100mm x 20mm, P5606, Sarstedt, Germany) was filled with RIPA buffer (SDS 0.1%, NaCl 150mM, IGEPAL CA-630 1%, deoxycholic acid 0.5%, and TRIS 50mM), and one embryo at a time was transferred with as little E3 medium as possible. Individual embryos were transferred into a 2ml safe lock reaction tube using a pipet set to 10μl and a pipet tip which was cut to avoid shearing. The embryos (a pool of five) were either stored at 20°C or processed directly. For lysis, a 3mm carbide bead and 50μl of RIPA buffer were added to the tubes and the five embryos were lysed with the TissueLyser LT (QIAGEN, Netherlands) for 5min at a frequency of 40Hz. Afterward, the samples were placed on ice for 30min and the lysate was diluted 1:3 by adding distilled water. The dilution was centrifuged at 4°C for 30min at full speed with a table top centrifuge (Centrifuge 5,427 R, Eppendorf, Germany), and the supernatant (about 130μl) was removed and transferred to a fresh reaction tube for storage at 20°C.

#### Protein Quantification

Protein concentration was determined using Bradford reagent. The protein samples were diluted 1:3 to reduce measurement interference with the RIPA buffer. Dilutions of 2mg/ml non-free fatty acid-free BSA (A7906, Sigma-Aldrich, United States) stock were used as standard. In a black 96-well microplate (655,096, Greiner Bio-One), 5μl of sample were added to 250μl Bradford reagent (B6916, Sigma), mixed and incubated for 5min at room temperature, and shielded from light. The absorbance was detected using an EnSpire Multimode Plate Reader (PerkinElmer, United States) at 595nm. All samples were measured in triplicates.

#### DNA Quantification

DNA concentration was determined using the Quant-iT™ PicoGreen® dsDNA Kits (P7589, Invitrogen, United States), following the manufacturer’s protocol (high range standard curve, 1μg – 0.01 μg). For the DNA standard curve, 2μg/ml of stock dsDNA solution was diluted in 1:3 RIPA buffer. The fluorescent signal was detected with a CLARIOstar plate reader (BMG Labtech, Germany) adjusted to the PicoGreen pre-set (top optic, excitation: 483–15, dichroic: auto 502.8, and emission: 530–30). All samples were measured in triplicates.

### Seahorse (XF96) Temperature

To adjust the XF96 Seahorse extracellular flux analyzer (Agilent, United States) to various temperatures, the desired environmental and tray temperature were set in the menu instrument/administration/temperature control. To achieve 18°C measurement temperature, the analyzer was placed in a temperature-controlled 5°C room for passive cooling. Notably, the analyzer was moved back to room temperature after the measurement to avoid condensation in the machine. For 23°C and 28°C assay temperatures, cooling of the Seahorse was supported by desk fans circulating cool room air into the vents. The analyzer maintained assay temperatures between 33°C and 45°C without any external support, while a thermal fuse prevented the use of the machine beyond 45°C by disabling the in-built heaters.

### Seahorse Measurements

#### Cartridge Preparation

The Seahorse cartridges (102,416, Agilent, United States) were hydrated by adding 200μl of XF calibrant solution (100,840, Agilent, United States) to the utility plate and incubated overnight at 37°C.

#### Sedation and Transfer to the Seahorse Well Plate

Tricaine (E10521, Merck, United States) was used to reduce embryo movement in the Seahorse well and to stabilize OCR, as previously shown by Raftery et al. (2017). The embryos were incubated in a petri dish containing 125mg/ml tricaine in E3 medium and then transferred into the Seahorse XF96 cell culture plates (101,085, Agilent, United States) with a 100μl cut pipet tip before adding 170μl of XF base medium with minimal DMEM (103,334, Agilent, United States) to each well. The central position of the embryos was checked with a microscope and corrected with a shortened Microloader™ pipet tip (EP5242956003, Eppendorf, Germany), if necessary. For analyzing the effect of sedation on the variability of OCR, tricaine was injected *via* port A of the XF96 cartridge.

#### Measurement Protocol

**Figure 1:**
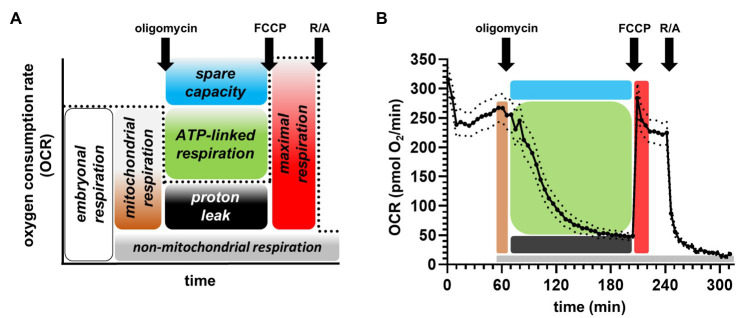
Partitioning embryonic respiration into bioenergetic modules. **(A)** General scheme depicting the analysis of the mitochondrial stress assay, showing the different respiratory modules. FCCP: carbonyl cyanide-p-trifluoromethoxyphenylhydrazone; R/A: rotenone and antimycin A. **(B)** Exemplary averaged trace measuring embryos at 28°C. The solid trace represents the mean, while the dotted lines depict the standard error of the mean.

The baseline of embryonic respiration was measured in 15cycles, with one cycle consisting of 1min mixing, 1min waiting, and 2min measuring. The last three cycle values before oligomycin addition were averaged to determine embryonic embryonal respiration. Oligomycin (O4876, Sigma-Aldrich, United States), injected *via* port A at a final concentration of 25μm to inhibit ATP synthase, served to determine respiration linked to ATP synthesis and to proton leak. Thirty cycles were required to establish steady-state rates at low temperatures, and the average of three lowest consecutive points was taken as value for proton leak. Subsequently, 8μm carbonyl cyanide-p-trifluoromethoxyphenylhy drazone (FCCP; C2920, Sigma-Aldrich, United States) was added (port B) to uncouple respiration and maximize substrate oxidation for eight cycles. The average of the three highest points determined maximal respiration. Finally, 1.5μm rotenone (R8875, Sigma-Aldrich, United States) and 1.5μm antimycin A (A8674, Sigma-Aldrich, United States) served to block mitochondrial respiration and determine non-mitochondrial respiration as the average of the three lowest consecutive points. This value was subtracted to receive mitochondrial respiration rates. ATP-linked respiration was calculated by subtracting proton leak respiration from basal respiration. Spare respiratory capacity was calculated by subtracting basal mitochondrial from maximal respiration. Coupling efficiency (CE) reports the fraction of mitochondrial respiration dedicated to ATP synthesis and is the quotient of ATP-linked/basal mitochondrial respiration. In cases, where proton leak respiration is negligibly low, or slightly negative values were received by subtraction of non-mitochondrial respiration, CE was set to 1. Figure 1 depicts a schematic measurement example of embryos exposed and measured at 28°C. Mitochondrial inhibitors and FCCP were dissolved in dimethyl sulfoxide and diluted with XF base medium (103,334, Agilent, United States) with minimal DMEM. The mitochondrial stress assay is a fatal experiment for the embryo. The embryos are considered alive during the measurement of basal respiration and may die during oligomycin treatment (~70min after start), since ATP synthase inhibition *via* oligomycin is irreversible (Wyatt and Buckler, 2004). Furthermore, FCCP and finally rotenone/antimycin A treatment are deleterious for the organism.

### Analysis of Embryo Heart Rate

Heartbeats per second of the transparent embryos were counted visually using the microscope. All embryos were removed from the water bath incubation simultaneously and placed in fresh 100mmx20mm petri dishes (P5606, Sarstedt, Germany) filled with E3 medium at RT. The embryos were anesthetized with five drops of 4gl^−1^ tricaine solution to reduce movement, since Raftery et al. (2017) demonstrated no impact of 75mg/l – 175mg/l tricaine treatment on heart rate within 2hours. Then, the embryos were aligned vertically and the heart rate was recorded for 15s with a four-digit hand-held tally counter (ENM, United States). The final heart rate was determined by counting temperature pre-exposed embryos on two different experimental days.

### Statistics

All data are presented either individually or as means ± SEM. Students *t*-test was applied to test for the difference of OCR of chorionated vs. dechorionated embryos. Ordinary one-way ANOVA followed by Tukey’s multiple comparisons test was applied to investigate the impact of sedation on OCR and the impact of temperature on protein/DNA content. Two-way ANOVA followed by Tukey’s multiple comparisons test was performed to test for differences in OCR in response to temperature pre-exposure and different assay temperatures. For differences in heart rate, ordinary one-way ANOVA was applied and followed by Dunnett’s multiple comparisons test, with the standard temperature of 28°C as control value. Values of *p*<0.05 were considered statistically significant. All statistical tests were performed using GraphPad Prism version 8.0.0 for Windows, GraphPad Software, United States.

## Results

### Analysis of Cellular and Mitochondrial Respiration

The well-established mitochondrial stress assay for extracellular flux analyzers was applied to measure the bioenergetics of the zebrafish embryos. Figure 1A depicts the scheme of the assay and how to calculate the values for the different modules of mitochondrial energy transduction upon the injections of compounds. ATP synthase inhibitor oligomycin served to partition basal mitochondrial respiration into ATP-linked and proton leak respiration, chemical uncoupler (FCCP) stimulates respiration to determine maximal respiration/substrate oxidation and spare respiratory capacity of the embryo. Finally, the respiratory chain inhibitors rotenone and antimycin A (R/A) enabled correction for non-mitochondrial respiration. A typical averaged respiratory trace of embryos maintained and measured at 28°C is shown in Figure 1B, and the criteria to average values for embryonic, proton leak, FCCP, and non-mitochondrial respiration are described in Material and Methods.

### Impact of the Chorion on Respiration

**Figure 2:**
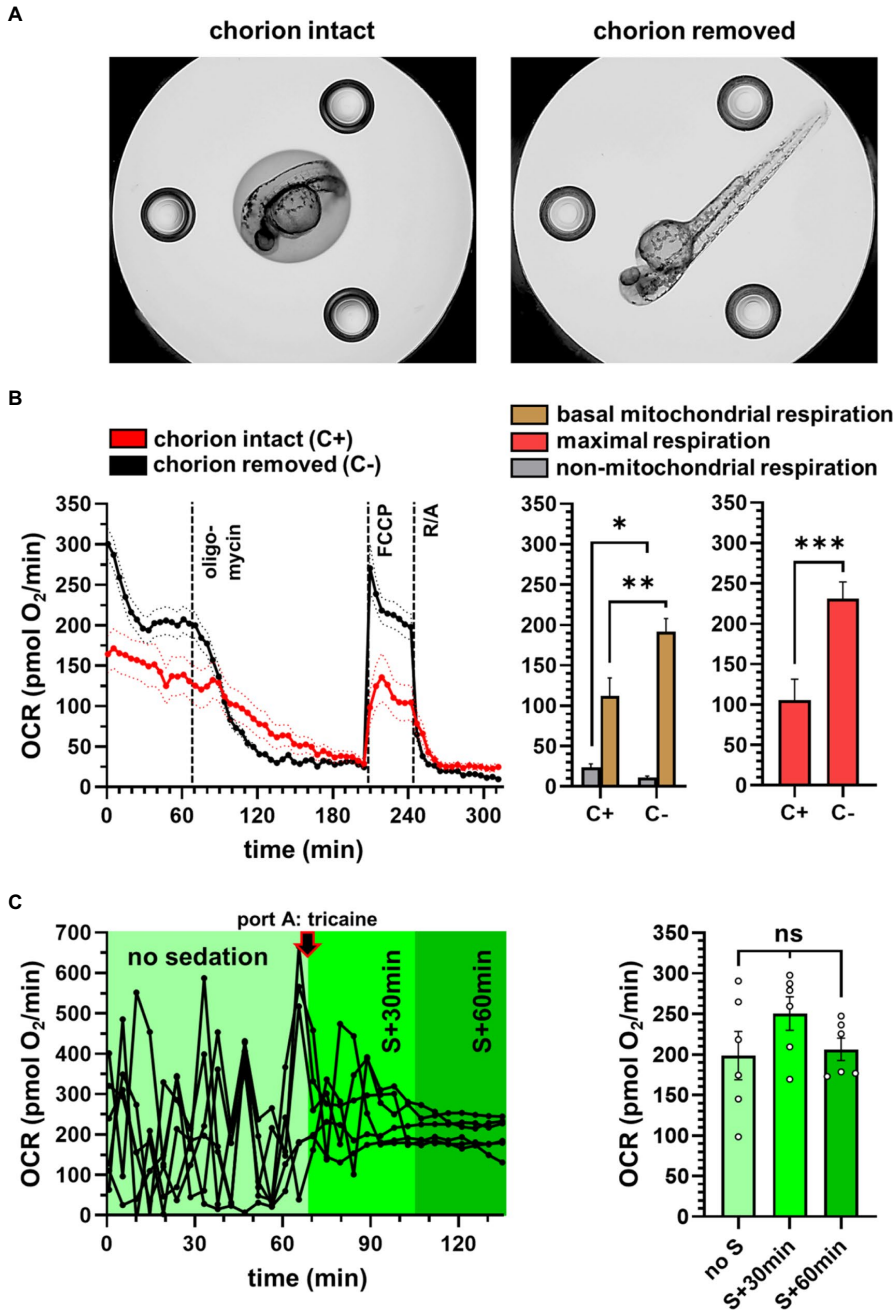
Experimental conditions to measure zebrafish embryos in the XF96 Seahorse. **(A)** Microscopic image of the zebrafish embryos in the Seahorse well, without (left) and with (right) chorion removal. **(B)** Effect of the chorion on OCR, showing reduced OCR with an intact chorion. Mean traces represent 36 wells for each group. **(C)** Effect of sedation on the respiratory traces. Respiratory variability was evaluated on individual traces using six individual embryos. S: sedation. ^*^p<0.05; ^**^p<0.005; ^***^p<0.0005.

Next, we assessed the impact of the chorion on respiration oneday after chorion removal. The microscopic images (Figure 2A) depict the embryos with and without choria, which were positioned centrally in the well to reduce variability between the respiratory traces. Furthermore, embryos with chorion appeared to be crushed after the experimental run, e.g., leaking of the yolk sack, while straightened embryos without the chorion appeared to be more intact (see supplemental images). In our hands, the chorion removal 24h prior measurement resulted in increased basal respiration and improved the response to FCCP (Figure 2B). Thus, chorion removal was used for all following experiments.

### Impact of Sedation on Respiration

Tricaine has been used previously (Raftery et al., 2017) to reduce the noise of the respiratory traces. We confirmed the positive effect of tricaine in our experiments, showing that the variability of the respiratory readouts was reduced (Figure 2C) and importantly, did not significantly change the average OCR value.

### Temperature Exposure and Morphological Consequences

**Figure 3:**
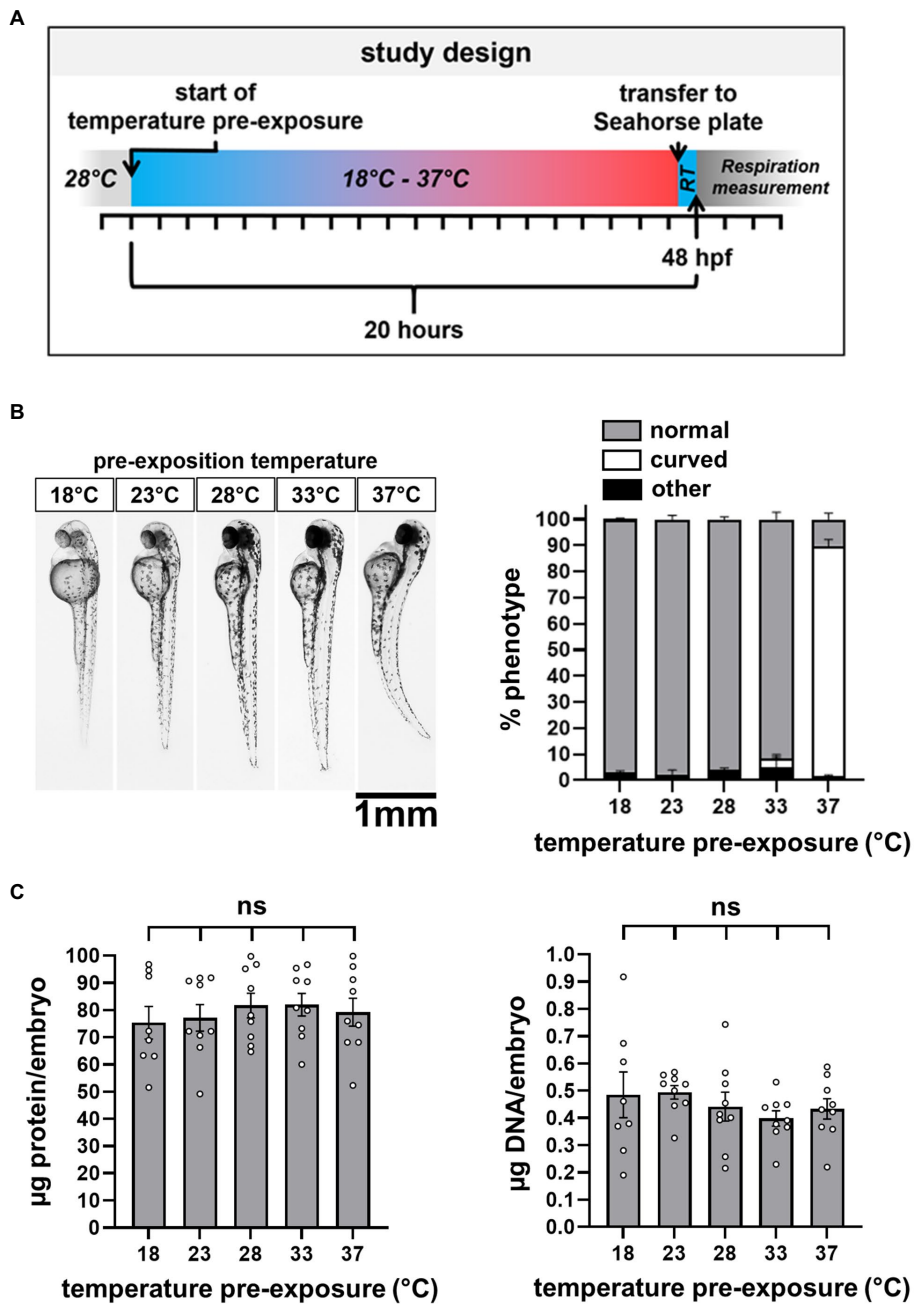
Study design and temperature-dependent changes in embryo morphology. **(A)** Short outline of the study design. A comprehensive outline can be found in the Supplementary Figure. RT: room temperature of 22±1°C. **(B)** Light microscopy to evaluate shape changes, categorized in normal, curved, and other. **(C)** Quantification of protein and DNA content per embryo.

Next, we exposed the embryos, previously maintained at 28°C, to medium temperatures of 18°C, 23°C, 28°C, 33°C, and 37°C for 20h, before moving them to the measurement well plate (Figure 3A and Supplementary Figure). The embryos showed pronounced changes in morphology toward a curved phenotype with exposure to 37°C (Figure 3B), prompting us to investigate changes in biomass in response to temperature exposure. We found that neither protein nor DNA content was significantly different between experimental groups (Figure 3C). Thus, we did not correct the OCR for biomass.

### Measurements of Embryonic Respiration at Different Temperatures

**Figure 4:**
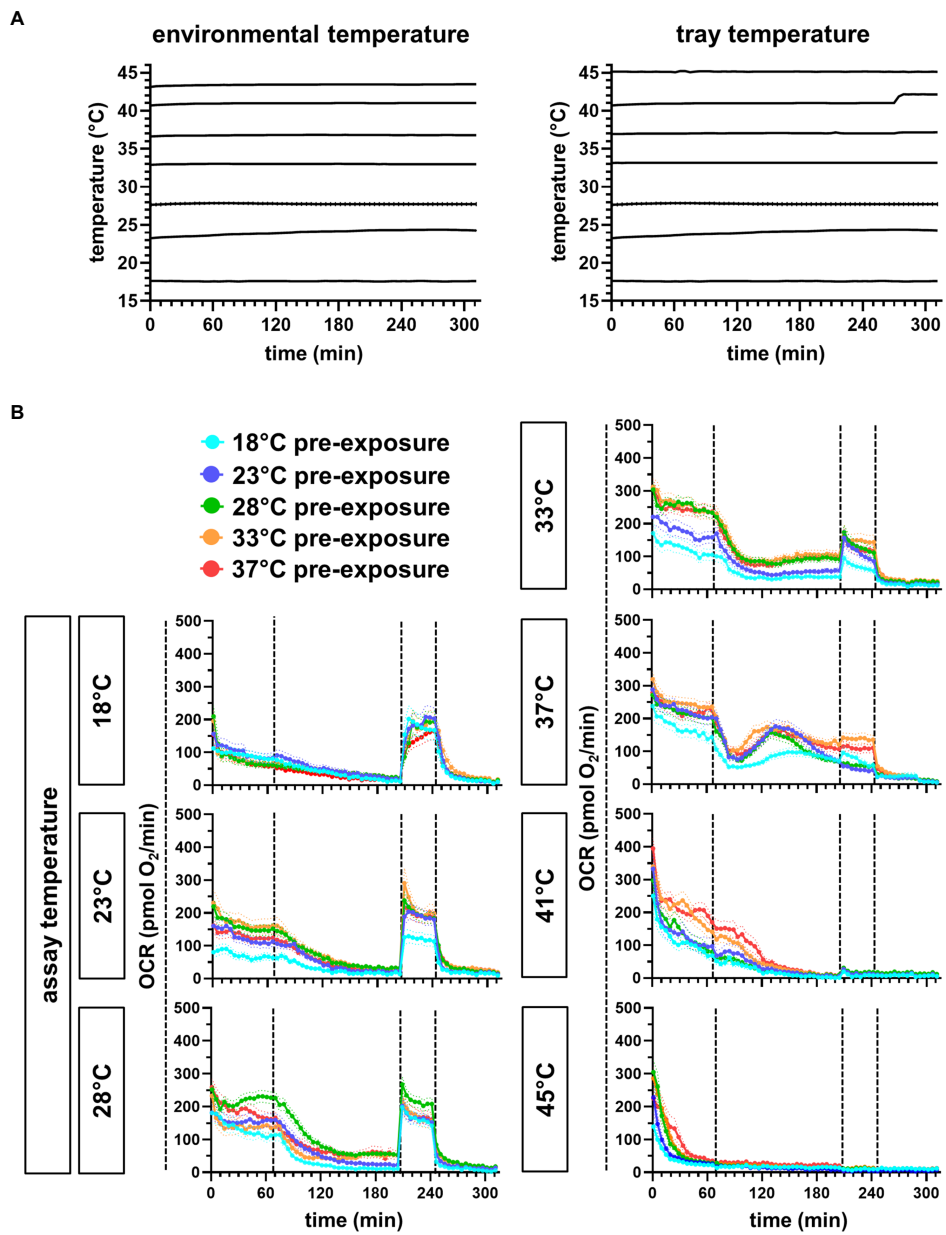
Continuous temperature registration of the XF96 Seahorse and respiration traces of differentially exposed embryos at different assay temperatures. **(A)** Instrument temperatures of the interior (environmental temperature) and of the tray, where the plate wells are located. **(B)** Pre-exposure temperatures are depicted in different trace colors. The mean trace is represented by n individual measurements (*n*=16 for 18°C, 41°C, and 45°C; *n*=32 for 23°C, 28°C, 33°C, and 37°C, measured on two experimental days).

First, we set up the XF96 in stable thermal environments to achieve stable measurement temperatures as described in Material and Methods. Temperatures registered in the tray, which reflect closest the measurement temperature, and the interior temperature of the instrument can be retrieved. Exemplified for one plate (Figure 4A), the temperature appeared to be quite stable over the measurement time. Overall, the OCR traces of differentially pre-exposed embryos at different assay temperatures reveal the increase of respiration rates with increasing temperatures and respiratory failure at higher assay temperatures (Figure 4B).

### Analysis of Respiratory Traces at Different Temperatures

**Figure 5:**
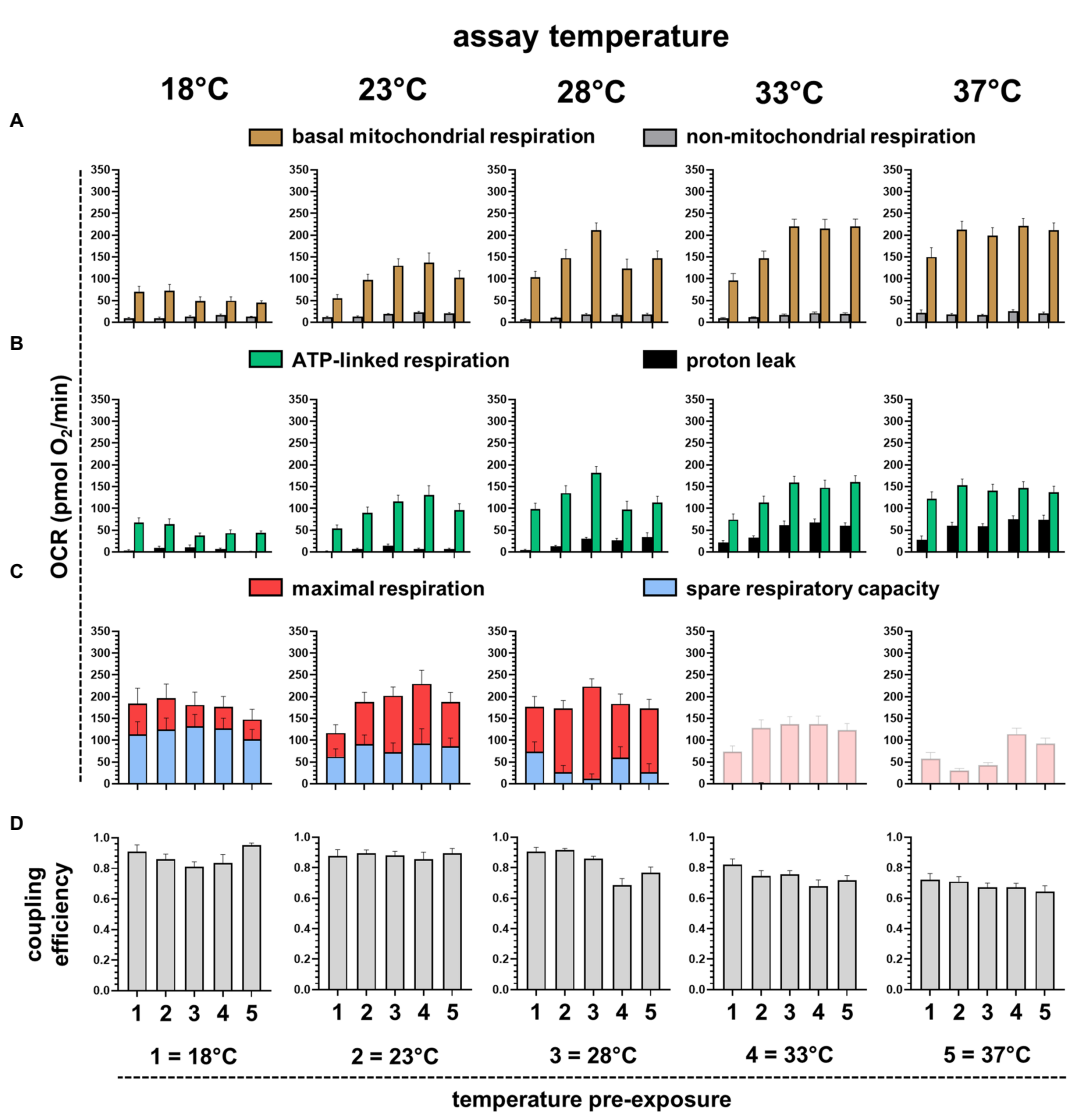
Analysis of different respiratory parameters of embryos pre-exposed to different temperatures and measured at different assay temperatures. **(A)** Mitochondrial (brown) and non-mitochondrial (gray) respiration determined with rotenone and antimycin A. **(B)** ATP-linked (green) and proton leak (black) respiration determined using oligomycin. **(C)** Maximal respiration (red) induced with FCCP and spare respiratory capacity (blue), determined by subtracting basal respiration. At 33°C and 37°C, FCCP did not induce respiration above basal respiration and thus, could not be further analyzed. **(D)** Coupling efficiency (CE) calculated as fraction of respiration dedicated to ATP synthesis (ATP-linked respiration) of basal mitochondrial respiration. Averaged values represent 16–32 individuals.

Next, we analyzed the respiration traces of the assay temperatures from 18°C to 37°C, partitioning respiration into functional modules as described above (Figure 1). Basal mitochondrial respiration increased with temperature up to 28°C and remained stable up to 37°C (Figure 5A). Non-mitochondrial respiration was low throughout the different measurement conditions. Proton leak rates were negligible up to 23°C but then increased with increasing assay temperature (Figure 5B), while ATP-linked respiration remained stable between 28°C and 37°C. Maximal respiration between 18°C and 28°C was stable, while spare respiratory capacity decreased simultaneously (Figure 5C). Given the decreasing effects of FCCP with increasing temperatures, the maximal respiration rates at 33°C and 37°C remained below basal respiration, presumably due to damage over time and thus, were highly confounded and not used for further analysis. Increasing proton leak and stable ATP-linked respiration should decrease the efficiency to convert nutrient energy to ATP, and this is seen as decreasing coupling efficiency (CE) with higher temperatures (Figure 5D). Notably, CEs were calculated for each fish individually and are therefore a powerful internally standardized parameter for individual mitochondrial efficiency.

### Mitochondrial Factors Potentially Limiting Metabolic Performance

**Figure 6:**
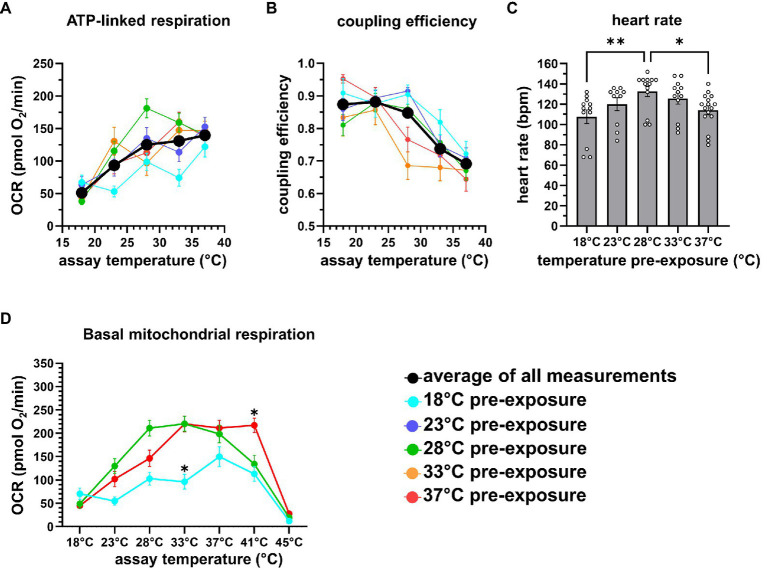
Temperature sensitivity of respiration. **(A)** Colored traces depict the averages of ATP-linked respiration of different temperature pre-exposures, the black solid trace represents the average of all measurements. **(B)** Colored traces indicate coupling efficiency of different pre-exposures, the black solid trace represents the average of all measurements. **(C)** Bar chart depicting the average of the heart rate (*n*=12–14) in response to temperature pre-exposure, measured at room temperature. **(D)** Basal mitochondrial respiration of 18°C, 28°C, and 37°C pre-exposed embryos is plotted vs. assay temperature, revealing lower respiratory activity of 18°C pre-exposed embryos, and the resilient respiration rates of 37°C vs. 28°C pre-exposed embryos at higher assay temperature. *p<0.05; **p<0.005.

To get insights into the mitochondrial limitation for systemic metabolism, we plotted selected mitochondrial respiration parameters against assay temperatures. Mitochondrial activity is low at 18°C assay temperature, but this is not caused by the limitation of substrate oxidation, as maximal respiration is not reduced and substantial spare respiratory capacity is available (Figure 5C). Therefore, low mitochondrial activity must be controlled by low ATP synthase activity, or more likely, by low cellular ATP turnover. Respiration linked to ATP turnover increases steadily with assay temperature, depicted in the temperature performance curve of ATP-linked respiration (Figure 6A). Coupling efficiency is stable up to 28°C assay temperature and then drops by about 0.15=15%, meaning that 15% more energy is lost as heat due to increased proton leak at temperatures above 28°C (Figure 6B). The lack of trustable FCCP rates at 33–37°C prevented us from analyzing limitations of substrate oxidation. Collectively, we would expect a decrease of metabolic performance at lower and higher temperatures. Using heart rate as an independent indicator of systemic metabolic rate (Figure 6C), we found peak rates for embryos pre-exposed to 28°C, while the heart rate was significantly decreased in embryos pre-exposed to 18°C and 37°C.

### Resilience of Embryonic Respiration to High Temperatures by Pre-exposure to Warm Conditions

Finally, we analyzed whether temperature pre-exposure improves temperature resilience of embryonic respiration at higher temperatures. Therefore, we plotted basal mitochondrial respiration measured between 18°C and 45°C of the cold (18°), normal (28°), and warm (37°C) pre-exposed embryos (Figure 6D). In 18°C pre-exposed embryos, oxygen consumption was generally lower. Respiration of embryos at 18°C and 28°C, the latter resembling the routine maintenance temperature, withstands assay temperatures up to 37°C. Embryos which were pre-exposed to 37°C, however, maintained higher basal respiration rates up to 41°C. At 45°C assay temperatures, respiration of all embryos collapsed immediately.

## Discussion

The life cycle bottlenecks, which represent the most temperature-sensitive life stages, are often not clearly defined (Dahlke et al., 2020). We decided to investigate the developing embryo as vulnerable bioindicator for temperature sensitivity and climate change, as we can apply Seahorse technology on whole embryos. The assessment of respiration rates in zebrafish embryos with the XF96 extracellular flux analyzer allows measuring systemic oxidative metabolism in a multi-well format, determining with various mitochondrial effector injections, which modules of mitochondrial energy transduction are changed in response to different treatments. These changes in oxygen consumption enable us to address distinct bioenergetic mechanisms. We investigated the effects of temperature pre-exposure and assay temperature on the mitochondrial performance of zebrafish embryos to receive mechanistic insights underlying systemic metabolism and its limits. We show that slow respiration in the cold is caused by low ATP turnover and is not limited by mitochondrial oxidative power, suggesting slow cellular metabolism. In the warm, basal mitochondrial respiration is stable before dropping above 37°C, paralleled by an increasing proton leak which becomes more impactful for the consumption of proton motive force and therefore, limits mitochondrial efficiency. Our experiments also show that the thermal window of stable embryonic respiration rates can be extended toward higher temperatures by pre-exposing the embryos to the warmth.

The plate-based respirometry of the XF96 analyzer can measure up to 92 individuals in parallel and imposes less shearing stress on the embryos as compared to chamber-based respirometry, which requires constant stirring. XF analyzers have been used for zebrafish bioenergetics in previous studies (Stackley et al., 2011; Shim et al., 2016; Raftery et al., 2017; Souders et al., 2018; Lee et al., 2019). In contrast to previous observations showing reduced respiration after chorion removal in islet capture plates of the XF24 system (Souders et al., 2018), we found that dechorionation of embryos enhanced respiration and responses to mitochondrial inhibitors and uncouplers in the XF96 system. Notably, our embryos were dechorionated at a later developmental stage of post-fertilization, which could explain discrepant results. However, physical damage on the embryo in the small respiratory chamber of about 2–3μm clearance also appeared to be visually absent when the chorion had been removed prior measurement. We confirmed that sedation with tricaine reduces variability of oxygen traces that may be caused by spontaneous activity of the embryos. The mitochondrial inhibitor concentrations were deduced from previous publications, where effective inhibition of the ATP synthase in 50 hpf embryos was shown with 25μm oligomycin (Gibert et al., 2013), maximal stimulation of substrate oxidation in 48 hpf zebrafish with 8μm FCCP, and full inhibition of complex I and III with 1.5μm rotenone/antimycin A (Lee et al., 2019). Some compound concentrations, however, may have to be systematically re-assessed in future studies, as, for example, temperature may alter the sensitivity to uncoupler reagents. FCCP treatment at high assay temperatures (33°C and 37°C) did not induce higher respiration rates above basal levels, indicating vulnerability of the embryos. Furthermore, some cellular functions of the embryos may decay during the experiments, e.g., due to ATP depletion after oligomycin injection, which may also confound uncoupler-induced respiration rates. These caveats can be overcome by either changing the uncoupling compounds and/or concentrations depending on temperature, by changing measurement times, or by directly injecting various compounds in the first port to split the mitochondrial stress assay into separate experiments. Similarly, further experimentation could be applied to exclude any deleterious effects of the sedative tricaine at different assay temperatures or in differently temperature-exposed embryos. We applied concentrations in our experimental set up that were either used in many publications by others, briefly checked for sensitivity, or which did not show obvious adverse effects (e.g., tricaine over time) during the conventional mitochondrial stress assay.

We chose to start exposure to different temperatures not before 24 hpf despite reducing the exposure time to 20h, as direct exposure of fertilized eggs to 33°C and 37°C would result in low survival rates, which are likely due to the impairment of gastrulation, a critical developmental step that is marked by blastoderm epiboly occurring at approximately 5.25 hpf (Kimmel et al., 1995). Maintaining the embryos on the standard temperature of 28.5°C ensured undisturbed onset of development and increased survival in the later temperature exposure experiments.

In the oxygen traces at 37°C assay temperature, we found reproducible increases of proton leak respiration 30min after oligomycin injection. The molecular nature of this increase remains unknown but could reside in apoptotic processes, which increase mitochondrial membrane permeability, such as permeability transition.

From all the OCR parameters, it transpires that embryonic metabolism is reduced at 18°C, reflected by decreased ATP-linked respiration. Notably, ATP-linked respiration did not further increase at higher temperatures, thus increasing the impact of the mitochondrial proton leak. We quantified the energetic efficiency to produce ATP by calculating coupling efficiency CE (Kabra et al., 2021). CE is the fraction of mitochondrial respiration linked to ATP synthesis (CE=ATP-linked respiration/basal mitochondrial respiration) and decreases from >0.85 to about 0.7 above 33°C assay temperature. Thus, the efficiency to convert nutrient energy to ATP decreases by about 15% and could negatively impact energy metabolism. These observations are in accordance with data of isolated mitochondria in ectotherms. For example, substrate oxidation in isolated fish muscle mitochondria increases with assay temperature (Guderley and Johnston, 1996), and proton permeability of isolated liver mitochondria increases with acclimation temperatures, as shown for the common carp (Jastroch et al., 2007) or the cane toad (Trzcionka et al., 2008).

Which mitochondrial parameters appear most important for metabolic performance in response to temperature? Using embryo heart rates as indicator of systemic metabolism, we found a bell-shape distribution over exposure temperatures, peaking around 28°C. Slower metabolism below 28°C is best reflected in the reduced ATP-linked respiration. At higher temperature above 28°C, CE is impacted by the proton leak, possibly limiting metabolic performance. Thus, these two parameters could be used to explain temperature phenomena on metabolic performance. Importantly, CE as internally standardized parameter can also be used in cross-study comparisons, as experimental differences of absolute values are eliminated.

We found that 37°C pre-exposed individuals expand their thermal window for stable mitochondrial respiration rates up to 41°C, demonstrating some capacity to expand tolerance of higher temperatures. This observation could be instrumental for judging the impact of shifting environmental temperatures, e.g., during global warming. At least during early development, pre-exposure to higher temperatures is beneficial.

This project explored the impact of temperature on mitochondrial bioenergetics. With these assays, we aim to investigate effects of genetically modified zebrafish to identify mechanisms that are causally linked to thermo-tolerance. Furthermore, the experimental protocol can be used to understand how environmental pollutants (e.g., heavy metal ions) interfere with the temperature-bioenergetics axis of an aquatic organism, also using other species.

Collectively, we show that the Seahorse extracellular flux analyzer platform can robustly assess mitochondrial function *in situ* and that the analysis of respiratory parameters could be integrated in modeling of temperature sensitivity of metabolic performance in small organisms.

## Data Availability Statement

The original contributions presented in the study are included in the article/Supplementary Material, and further inquiries can be directed to the corresponding author.

## Ethics Statement

The animal study was reviewed and approved by the Stockholm North Ethical Committee with the permit number 14049–2019.

## Author Contributions

ER performed all experiments and analyzed the data. ER and MJ wrote the manuscript. MJ conceptualized the study. All authors have edited and proof-read the manuscript.

## Funding

This study is supported by the Swedish Research Council (Vetenskapsrådet; 2018–03472 to MJ).

## Conflict of Interest

The authors declare that the research was conducted in the absence of any commercial or financial relationships that could be construed as a potential conflict of interest.

## Publisher’s Note

All claims expressed in this article are solely those of the authors and do not necessarily represent those of their affiliated organizations, or those of the publisher, the editors and the reviewers. Any product that may be evaluated in this article, or claim that may be made by its manufacturer, is not guaranteed or endorsed by the publisher.
