# Supplementary material for: Plate-Based Respirometry to Assess Thermal Sensitivity of Zebrafish Embryo Bioenergetics in situ
Source: Front Physiol. 2021 Sep 21;12:746367. doi: 10.3389/fphys.2021.746367 (PMC8491625; doi:10.3389/fphys.2021.746367)
Supplement: Supplementary file 1 [file Data_Sheet_1.pdf]

Supplementary Material for

Plate-based respirometry to assess thermal sensitivity of zebrafish embryo bioenergetics *in situ*

Erik Rollwitz[1], Martin Jastroch[1*]

[1] Department of Molecular Biosciences, The Wenner-Gren Institute, The Arrhenius Laboratories F3, Stockholm University, SE-106 91 Stockholm, Sweden

Supplemental figure. (**A**) Light microscopy images of zebrafish embryos in the Seahorse cell culture well plate after the measurement. Left image: embryo without chorion removal shows signs of damage, indicated by the arrow pointing to fat droplets, presumably from the yolk sack. Right image: dechorionated embryo (**B**) Extended scheme of the study design.

A

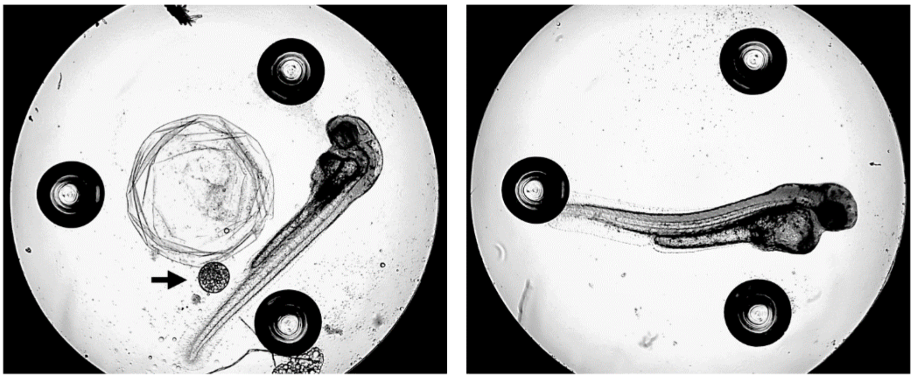

B

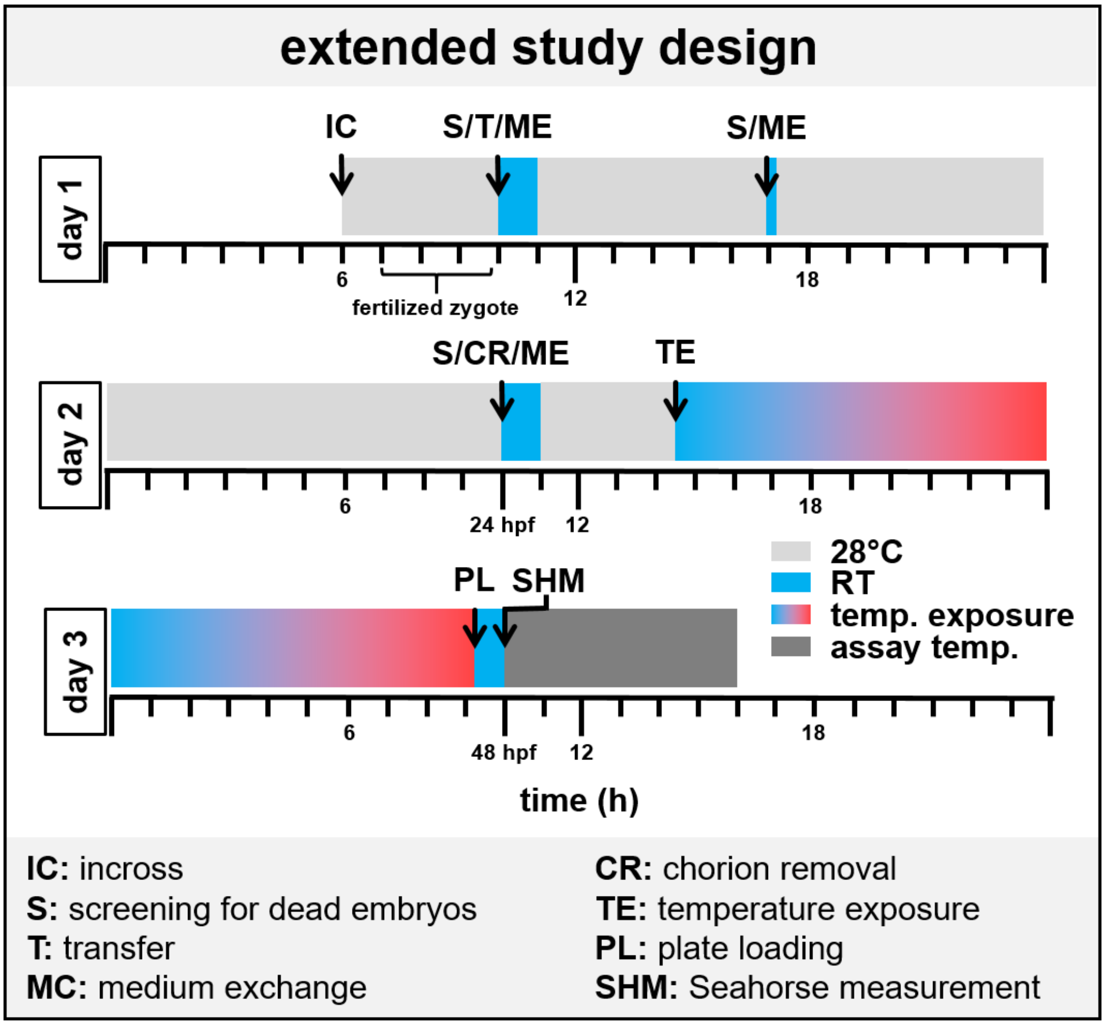
extended study design
IC
S/T/ME
S/ME
day 1
6
fertilized zygote
12
18
S/CR/ME
TE
day 2
6
24 hpf
12
18
28°C
RT
temp. exposure
assay temp.
PL
SHM
day 3
6
48 hpf
12
18
time (h)
IC: incross
S: screening for dead embryos
T: transfer
MC: medium exchange
CR: chorion removal
TE: temperature exposure
PL: plate loading
SHM: Seahorse measurement
